# A scoping review of unexpected weight loss and cancer: risk, guidelines, and recommendations for follow-up in primary care

**DOI:** 10.3399/BJGPO.2024.0025

**Published:** 2024-11-13

**Authors:** Javiera Martinez-Gutierrez, Lucas De Mendonca, Philip Ly, Alex Lee, Barbara Hunter, Jo-Anne Manski-Nankervis, Sophie Chima, Deborah Daly, George Fishman, Fong Seng Lim, Benny Wang, Craig Nelson, Brian Nicholson, Jon Emery

**Affiliations:** 1 Department of General Practice and Primary Care, The University of Melbourne, Melbourne, Australia; 2 Centre for Cancer Research, University of Melbourne, Melbourne, Australia; 3 Department of Family Medicine, School of Medicine. Pontificia Universidad Católica de Chile, Santiago, Chile; 4 Data Connect, Victorian Comprehensive Cancer Centre, Melbourne, Australia; 5 The Daffodil Centre, The University of Sydney, a joint venture with Cancer Council NSW, Sydney, Australia; 6 Primary Care and Family Medicine Department., Lee Kong Chian School of Medicine. Nanyang Technological University., Singapore, Singapore; 7 Primary Care Collaborative Cancer Clinical Trials Group (PC4), Melbourne, Australia; 8 Singapore Primary Care Cancer Network (SPriNT), Singapore, Singapore; 9 Department of Family Medicine, National University Health System, Singapore, Singapore; 10 Western Health Chronic Disease Alliance, Western Health Melbourne, Victoria, Australia; 11 Department of Medicine – Western Health, The University of Melbourne, Melbourne, Australia; 12 Nuffield Department of Primary Care Health Sciences. University of Oxford, Oxford, UK; 13 The Primary Care Unit, University of Cambridge, Cambridge, UK

**Keywords:** Cancer, Prevention, Screening, General practitioners, Primary healthcare

## Abstract

**Background:**

Cancer diagnoses often begin with consultations with GPs, but the non-specific nature of symptoms can lead to delayed diagnosis. Unexpected weight loss (UWL) is a common non-specific symptom linked to undiagnosed cancer, yet guidelines for its diagnostic assessment in general practice lack consistency.

**Aim:**

To synthesise evidence on the association between UWL and cancer diagnosis, and to review clinical guidelines and recommendations for assessing patients with UWL.

**Design & setting:**

Systematic search and analysis of studies conducted in primary care.

**Method:**

Four databases were searched for peer-reviewed literature from 2012 to 2023. Two reviewers conducted all the steps. A narrative review was conducted detailing the evidence for UWL as a risk factor for undiagnosed cancer, existing clinical guidance, and recommended diagnostic approach.

**Results:**

We included 25 studies involving 916 092 patients; 92% provided strong evidence of an association between UWL and undiagnosed cancer. The National Institute for Health Care and Excellence (NICE) Cancer Guideline in the UK was frequently cited. General suggestions encompassed regular weight monitoring, family history, risk factor evaluation, additional signs and symptoms, and a comprehensive physical examination. Commonly recommended pathology tests included C-reactive protein (CRP), complete blood count, alkaline phosphatase, and thyroid-stimulating hormone. Immunochemical faecal occult blood test, abdominal ultrasound, and chest X-ray were also prevalent. One large cohort study provided age, sex, and differential diagnosis-specific recommendations.

**Conclusion:**

This evidence review informs recommendations for investigating patients with UWL and will contribute to a computer decision support tool implementation in primary care, enhance UWL assessment, and potentially facilitate earlier cancer diagnosis.

## How this fits in

Unexpected weight loss (UWL) is a common non-specific symptom linked to undiagnosed cancer, yet guidelines for its diagnostic assessment in general practice lack consistency. A systematic review found an association with ten types of cancer in primary care. Gastro-oesophageal, colorectal, lung, pancreatic, prostate, and renal tract cancers were the most frequently studied. Common tests like C-reactive protein (CRP), raised neutrophils, and raised platelets may be useful to identify people at risk of cancer.

## Introduction

Patients with cancer often initiate their healthcare journey by consulting GPs about their symptoms. Diagnosing cancer in primary care poses significant challenges, as patients often present with less severe and non-specific symptoms.^1^ The presence of non-specific symptoms when a patient first presents to the GP can lead to delays in cancer diagnosis, contributing to increased mortality across various cancers.^2^ In a study conducted in Victoria, Australia,^3^ it was revealed that 34% of patients had three or more GP consultations for cancer-related symptoms before being referred to a specialist. The likelihood of multiple visits varied by cancer type: patients with pancreatic cancer or myeloma, presenting non-specific symptoms, had a higher probability of multiple visits than those with more specific symptoms like breast cancer or melanoma. Timely investigations and referrals in primary care are crucial, emphasising the need to identify patients at higher risk of undiagnosed cancer.^3^

UWL exemplifies a non-specific cancer presentation, posing a clinical challenge due to its various potential causes when isolated.^4^ Studies have associated UWL with 10 different types of undiagnosed cancer, its predictive value in male and female patients over 60 years exceeds 3%,^5^ which warrants further investigation according to international guidelines.^6^ Three per cent is also comparable to more specific clinically recognised 'red flag' symptoms such as rectal bleeding for colorectal cancer (2.4%), and haemoptysis in lung cancer (2.4–4.5%).^7^

Clinical recognition of UWL in primary care can be challenging. Several factors may contribute to this phenomenon including a lack of consensus on its definition,^4^ its non-specific nature,^4,8,9^ inconsistent weight measurements in general practice,^10^ concerns about patient sensitivity,^11^ and possibly limited community awareness of its clinical significance. Rao *et al* conducted a retrospective review of electronic medical records (EMR) including patients with a recorded weight loss of 5–10% in 6–12 months. They found that only 21% of UWL cases were correctly recorded as UWL in the EMR.^12^

UWL is defined as a loss of ≥5% in body weight in 6–12 months, not explained by medical treatment, known health conditions, or changes in diet or physical activity, with varying quantitative cut-offs in research.^4,5^ Nevertheless, different quantitative cut-offs can also be found in clinical research of up to 10% of body weight loss.^4^ Terminology is also varied in the literature, which adds another challenge in creating clinical standards: *unexpected*, *unintended*, *unintentional,* and *unexplained* are all terms commonly used to represent the condition.^4,13,14^

UWL is commonly presented as a red flag symptom in cancer guidelines worldwide.^6,15–18^ The National Institute for Health and Care Excellence (NICE) guidelines in the UK recommend urgent cancer investigation in patients with a risk of cancer above 3% and to consider primary care testing when higher than 2%.^6^ However, as evidence increases, in clinical practice, adherence to these guidelines is variable, and guidance on UWL as a risk factor for undiagnosed cancer and appropriate follow-up remains general and not tailored to primary care.^6^

The aim of this review was to summarise the latest evidence regarding the association between UWL and cancer diagnoses and identify recommendations for UWL investigation and follow-up for primary care patients at risk of cancer. This evidence will be used to inform the development of a Clinical Decision Support Systems (CDSS) for use in Australian general practice.

We provided an overview of three key questions in relation to UWL as a risk factor for cancer:

1. What is the current evidence regarding the association between UWL and cancer?
2. Which cancer guidelines are being cited regarding follow-up of patients with UWL?
3. What is current evidence-based follow-up for patients with UWL at risk of cancer in primary care?

## Method

The Preferred Reporting Items for Systematic Reviews and Meta-Analyses (PRISMA) criteria guided our narrative scoping review.^19^ Covidence^®^ review software facilitated title and abstract screening, full-text review, and data extraction.

### Search strategy

Our search strategy, detailed in Supplementary Table S1, incorporated MeSH headings and word variations for 'unexpected weight loss' (for example unexpect*, or unpredict*, or unexplained, or uninten*, or sudden weight loss), 'cancer', 'risk factor', and 'guidelines'. We did not restrict our search to primary care, so our inclusive approach considered diverse reviews providing valuable information. We searched MEDLINE, EMBASE, Cochrane Central Register of Controlled Trials (CENTRAL), and Web of Science (Clarivate). Supplementary searches included manual reviews, citation tracking, and expert recommendations. We included studies published between 30 January 1 2012 and April 2023 (approximately the last 10 years), given our focus on the current evidence of associations and recommendations for UWL as a cancer symptom.

### Inclusion and exclusion criteria

We included peer-reviewed studies addressing the association between cancer and UWL, cancer guidelines involving UWL, and follow-up recommendations such as the ordering of appropriate laboratory and diagnostic tests that are examples of evidence-based follow-up of patients with UWL that have also been included in clinical practice guidelines. As the aim of this review is to synthesise the current evidence on UWL as a risk factor for cancer, no restrictions were made on study type; we included all relevant original research, and systematic and narrative reviews. Exclusion criteria comprised studies on children (<18 years), case reports, grey literature, and conference abstracts. Screening and full-text review involved two independent reviewers from a pool of three (JMG, PL, LDM) with conflicts resolved through consensus or third-party opinion (JE).

### Data collection and analysis

Using a standardised template (Supplementary appendix 2), data extraction included study characteristics, participant details, comorbidities, cancer types, UWL associations, guidelines, general recommendations, follow-up with laboratory results, and other tests. Due to methodological diversity, data were initially extracted and analysed separately, and later combined into a comprehensive narrative synthesis. The report provided summary descriptive statistics to convey the multifaceted nature of the studies.

## Results

### Study characteristics

**Figure 1.**
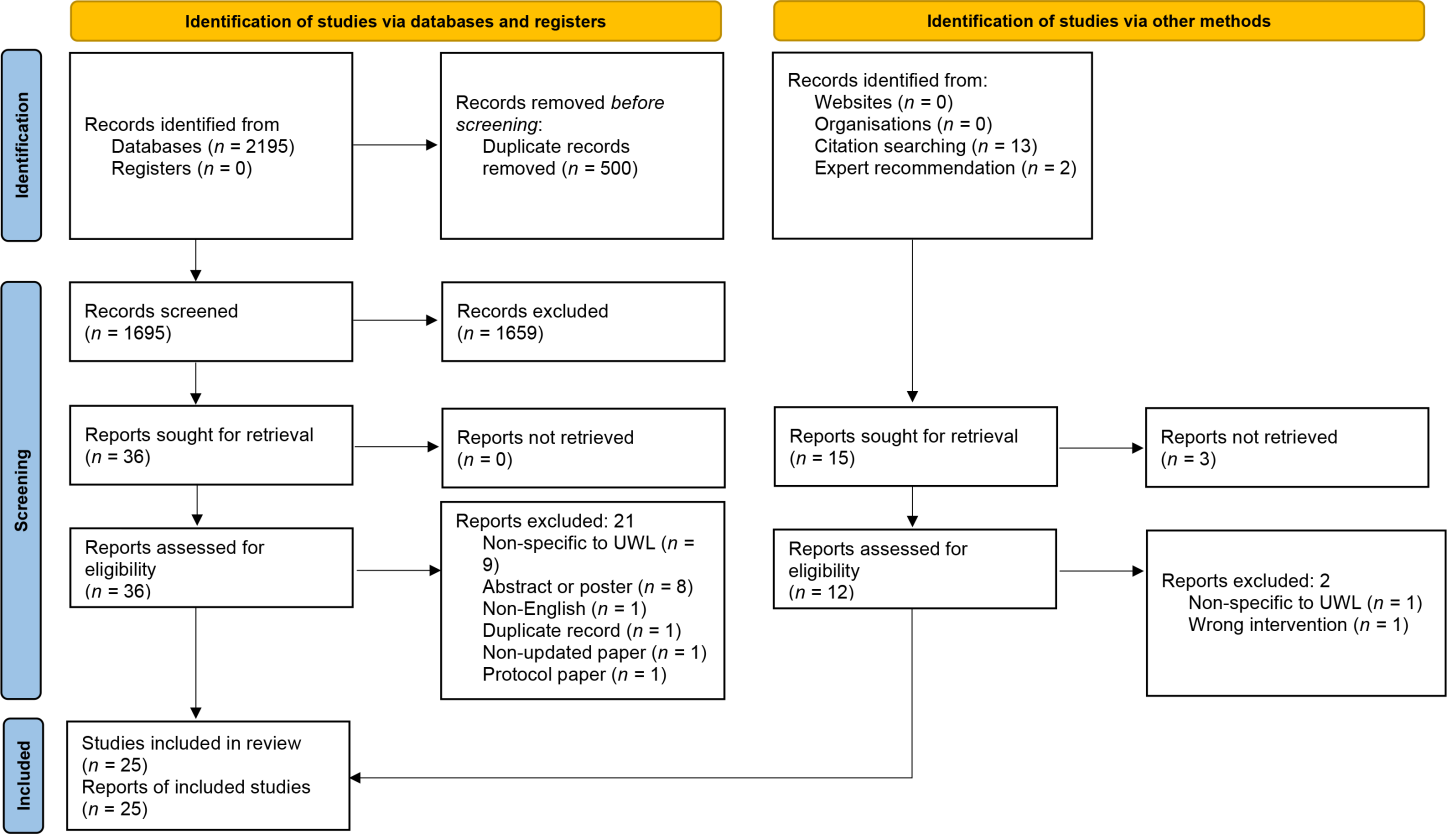
PRISMA flowchart.^19^ UWL = unexpected weight loss.

We included 25 studies in our analysis. Selection of studies is represented in Figure 1.

Out of the 25 analysed studies, 60% were primary studies and 40% were reviews. Primary studies were conducted predominantly in the USA and the UK, with additional contributions from Romania, Singapore, and Spain. Cohort studies constituted 60% of primary studies,^8,13,20–25^ followed by diagnostic accuracy studies (in the context of UWL, diagnostic accuracy studies are used to calculate the positive predictive value of UWL for cancer) (20%),^26–28^ and case-control studies (13%).^29,30^ Settings varied, including primary care (32%),^8,12,13,24,26–28,31^ hospitals (24%),^20–23,29,30^ and general population cohort (4%).^25^ A total of 916 092 patients were included, with a median of 2677 participants per study (123–365 275).

Encompassing participants aged 18–100 years, with a median age of 60 years or older in 48% of studies.^8,9,13,20–23,25–27,29,32^ Women constituted 60% of the total patients.

The seven narrative reviews^4,9,14,30,33–35^ and three systematic reviews^5,36,37^ conducted in the USA, UK, Netherlands, and Germany, provided a comprehensive overview of UWL and cancer.

It is relevant to note that of the 25 studies analysed, eight were conducted by Nicholson and their team at the University of Oxford.^5,8,13,24,26–28,33^ More information on study characteristics can be found in Supplementary Tables S2 and S3.

### Association between UWL and cancer

Strong evidence supports UWL as a risk factor for cancer across diverse populations, both in general practice and in patients with cancer.

Of the 23 studies exploring this association, 61% investigated UWL’s link to multiple cancers.^4,5,13,14,20–22,24,26–28,30,31,33^ Gastro-oesophageal, colorectal, lung, pancreatic, prostate, and renal tract cancers were the most frequently studied.

The prevalence of UWL varied from 5%^12^ to 33%^22^ (a median of 22%). Regarding the prevalence of cancer compared to other common causes of UWL, Withrow *et al* investigated a retrospective matched cohort of over 70 000 primary care patients. The study ranked the most frequent diagnosis in patients with UWL in primary care for 12 different conditions related to weight loss. For men aged 60–79, cancer was the most common condition diagnosed in patients with and without UWL, while in women with UWL in the same age group, cancer was the third most common diagnosis after depression and thyroid disorders. For men aged over 80, cancer was also the most common condition diagnosed.^8^

In the studies reviewed, the strength of association was well documented.^24,31^ Nicholson *et al*’s studies demonstrated the positive predictive value (PPV) of UWL, revealing increased cancer risk with age and additional risk factors.^13,24^ Increased hazard and odds ratios, and prevalence in patients with pancreatic cancer underscored the robust association.^25,32^

As an example, Nicholson *et al* identified men over 50 years old and people who smoked (currently or in the past) to be the populations at highest risk of cancer with PPVs over the required threshold for investigation of 3%.^26^ In a retrospective cohort analysis, the calculated PPVs per age range were: 0.58 for 40–59 years (0.5–1.16 calculated PPV range); 2.65 for 60–79 years(2.20–3.16); 2.99 for >80 years (2.35–3.75), which demonstrated that risk of cancer in patients with UWL increased with age. The probability also increased (to over 6%) if any of these individual tests were present: low albumin, raised CRP, raised neutrophils, or raised platelets.^27^ Probability also increased if UWL was paired with a combination of such tests.^28^

Interestingly, when calculating temporal associations, one study showed that the strength of the association between UWL and cancer decreased after 6 months of the UWL visit.^13^

A more detailed description of prevalence and other risk measures in the included primary studies are summarised in Supplementary Table 4.

Of the three systematic reviews identified, two reported UWL patients to have an increased likelihood of a cancer diagnosis.^5,36^ The third review did not provide evidence of an association between UWL and cancer, yet included guidelines related to UWL and its risk of cancer, and provided recommendations for follow-up, thus fitting our inclusion criteria.^37^

In a systematic review of primary care studies, Nicholson *et al* performed a meta-analysis of prospective cohort studies using EMR data to show an increased risk of cancer in patients with evidence of UWL in their EMR compared to patients without a recorded UWL. Importantly, UWL was associated with 10 cancers in primary care: prostate, colorectal, lung, gastro-oesophageal, pancreatic, non-Hodgkin’s lymphoma, ovarian, myeloma, renal tract, and biliary tree.^5^

The pooled sensitivity and specificity for UWL found were 14% and 97% for colorectal cancer respectively.^5^ The same parameters for pancreatic cancer, were 13% and 99% respectively. Data on efficacy were possible only for these two types of cancer because of their higher prevalence. In general, the PPV exceeded the 3% threshold for cancer investigation suggested by the UK guidelines in patients with UWL over aged 60 years in both males and females. Importantly, patients with weight loss were described as 1.6 times to 12.5 times more likely to have cancer than a patient without weight loss.^5,36^

All narrative reviews mentioned evidence of the association of UWL with cancer. In general, the risk associated with UWL was higher and presented wider intervals than the systematic reviews. Reported prevalence ranged from 6% to 37%. This may be explained by the fact that the settings in the narrative reviews were not clearly defined.^4,9,14^

Studies reported the risk increased when UWL co-occurs with other cancer symptoms.^33,34,36^ UWL was the second strongest predictor of cancer after other classic cancer symptom presentations, such as post-rectal bleeding for colorectal cancer, haemoptysis in lung cancer, and jaundice for pancreatic cancer.^33^

A comprehensive summary by study type, cancer sites, and other measures is reported in Supplementary Table S5.

### Guidelines including UWL as a risk factor for cancer

Forty per cent of studies referenced guidelines regarding follow-up of UWL. The most cited guidelines were the NICE guidelines for suspected cancer referral (70%).^6^ These guidelines contain pathways for investigation by symptom, including UWL; the most common reason for referring to them was their recommended 3% threshold for investigation.^6^

Two studies reviewed international guidelines including UWL as a red flag symptom for malignancy.^35,37^ Verhagen *et al* aimed to *'identify and descriptively compare the red flags endorsed in guidelines for the detection of serious pathology in patients presenting with low back pain to primary care'.* The authors reviewed 21 guidelines globally. Weight loss and low-back pain as a combination were included under consideration of 'malignancy' in Australian, Canadian, Finnish, French, German, Italian, and Dutch guidelines.^35^

The second study conducted by Van Melle *et al* reviewed current guidelines for the use of immunochemical faecal occult blood test (iFOBT) in symptomatic patients as a predictor of colorectal cancer in primary care. The Spanish, UK, and the Australian guidelines recommend iFOBT as part of the diagnostic assessment of patients with UWL without other high-risk symptoms suggesting a specific cancer site.^37^

### Recommendations for follow-up of UWL at risk of cancer

Sixty per cent of studies provided recommendations for follow-up of UWL. We classified recommendations as 'general', 'laboratory tests and imaging tests', and targeted recommendations by age, sex, and probable differential diagnosis.

#### General recommendations

General recommendations for follow-up of patients with UWL were provided by 44% of studies. Recommendations from reviews included a complete history and physical examination to identify other signs and symptoms that may lead to determination of causality. Perera *et al* recommended that a minimal history should consist of: associated symptoms, medication, dietary supplements and substance use, mood and cognition, diet, and psychological factors.^14^ Wong *et al* added assessment of cardiac, respiratory, and gastrointestinal symptoms, systemic signs of infection or malignancy, and evaluation of mental health symptoms such as anxiety, depression, and evidence of substance abuse.^4^ Other examinations included assessment of the oral cavity and dentition and examination for heart, lung, gastrointestinal, or neurological abnormalities.^9^

All studies recommended physical examination should always include body weight measurement. Two studies mentioned intervals of weight measurements by general practices, recommending 'frequent measurements',^24,30^ however, no specific intervals for weight measurements were provided.

Two studies in this review provided evidence in patients with pancreatic cancer suggesting that the risk of pancreatic cancer and mortality increases the higher the weight loss.^25,30^ However, there is insufficient evidence regarding the specific amount of weight loss over a certain period and how it correlates with the likelihood of a cancer diagnosis in primary care.^33^ In this review, only Wong *et al* tailored their recommendations to the amount of weight loss. They recommend 'using clinical judgement' to guide investigation in patients with weight loss <5% or longer than 6–12 months.^4^

### Laboratory investigations and images

The proposed baseline investigation consisted of complete blood count, basic metabolic panel, liver function tests, thyroid function tests, CRP, erythrocyte sedimentation rate, fasting glucose, protein electrophoresis, ferritin, urinalysis, HIV, calcium, lactate-dehydrogenase, FOBT, and PSA, all of which are readily available in most high income countries.^4,9,33^

As previously mentioned, several studies by Nicholson *et al* ^13,27,28^ tested laboratory test results within three months prior and one month after the index clinical appointment related to UWL as predictors of malignancy. PPVs increased to >6 if any of these common tests were abnormal: raised CRP, neutrophils, or platelets and low albumin. Interestingly, the findings suggested that simple risk scores including age, sex, and these primary care blood tests could enhance cancer risk stratification of UWL by the GPs. CRP was found to have the highest area under the curve as an individual marker (0.76 [95% confidence interval {CI} = 0.73–0.79) with 71% sensitivity and 80.5% specificity for cancer within 6 months of first presentation of UWL in primary care followed by raised neutrophils (0.64 [CI = 0.61–0.67]). Raised platelets showed a positive likelihood ratio of over 5 (the probability of having raised platelets is five times higher in a cancer patient than in a patient without cancer).^27^ A positive likelihood ratio of 5 is usually considered a good rule-in test.^38^

Furthermore, a cohort study provided insights into the intervals for investigation of UWL in primary care: for patients with an initial normal investigation, a watchful waiting approach may be appropriate with regular evaluations for new symptoms or signs of malignancy in the 3 months following the initial UWL appointment. This recommendation was based on the observation that the risk for malignancy dropped significantly after 3 months of the index appointment of UWL.^7^ This simple rule can guide selection of patients who warrant urgent referral and more extensive investigations from others for whom a 'watchful waiting' approach would be adequate.

**Figure 2.**
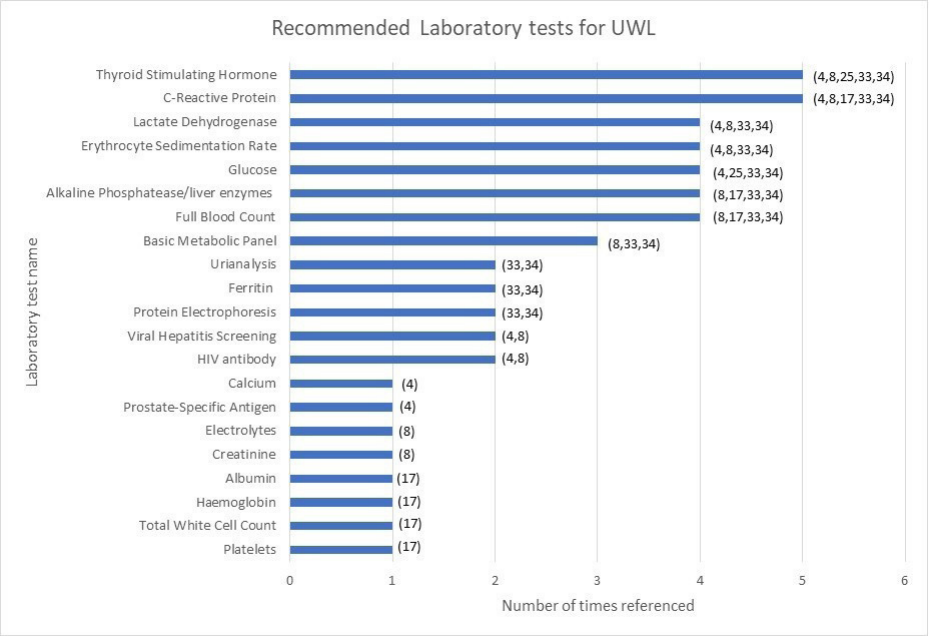
Recommended tests ranked by frequency of the recommendation.

A summary of the most frequently recommended tests can be found in Figure 2.

Initial imaging should consist of a chest radiography^4,9^ with additional imaging (such as ultrasound or computed tomography of the abdomen) to be ordered according to clinical judgement.^4,9,33^ Wong *et al* also recommended age-appropriate testing for breast, colorectal, and cervical cancer.^4^

### Targeted recommendations according to age, sex, and differential diagnosis

One systematic review and meta-analysis by Nicholson *et al* concluded that patients older than 60 years should be promptly evaluated after UWL due to their increased risk of cancer, but no specific recommendations on the type of follow-up were provided.^5^

Withrow *et al* studied a primary care cohort of over 70 000 patients. They ranked 12 serious conditions associated to UWL and provided recommendations tailored by the prevalence of conditions in specific age groups.^8^

A summarised version of all recommendations is shown on Supplementary Table S6.

## Discussion

### Summary

Despite being a well-known cancer symptom, UWL’s significance is often underestimated, with only 21% recognition by clinicians, as evidenced by Rao *et al*’s cohort study in the USA.^12^

The surge in research on UWL in general practice, particularly in the last 5 years, fueled by linked primary care databases, provides more robust evidence and insights into UWL trends and its association with cancer.

While older narrative reviews associated UWL with cancer, this had not been specifically studied in primary care patients. Nicholson *et al*’s body of work in primary care patients has been pivotal. Identifying associations of UWL and ten types of cancer, and specific populations at higher risk, such as men aged over 50 who currently smoke or formerly smoked, emphasised the importance of investigating UWL promptly.^24^

Our review found evidence that the strength of the association between UWL and cancer is most pronounced in the first 3–6 months after recognition, likely due to late-stage cancer presentations. After this period, the association decreases, suggesting alternative non-serious causes for UWL. Elevated CRP, increased neutrophils, and raised platelets are associated with increased risk of cancer.^27,28^

### Strengths and limitations

We used a systematic approach to review existing literature to answer the three research questions. Recognising that scientific literature might not encompass all cancer guidelines, our search focused on academic publications. Limited accessibility to recommendations from professional societies and international consortia, especially those not published academically, remains a constraint. Notably, the search in the International Guidelines Library yielded three irrelevant results.^39^ Acknowledging the existence of over a hundred guidelines worldwide, the review prioritised academic literature, acknowledging that summarising every guideline was beyond its scope.^15^

All studies included in this review originated from high-income countries and were written in English. As data usage and digital technologies evolve, future research should strive to include information from middle- and low-income nations, promoting a more comprehensive understanding of the global scenario.

### Comparison with existing literature

Although various tests according to clinical presentations are recommended, evidence shows that simple tests available in primary care can be markers of cancer risk. This is particularly the case for raised CRP, neutrophils and platelets, and low albumin.^24,27,28^ Bailey *et al* has found similar associations between raised platelet counts and cancer in primary care.^40,41^

iFOBT has been widely accepted as a good screening test for colorectal cancer.^42,43^ This review found that it can also be used in symptomatic patients (UWL as well as abdominal symptoms) to rule out colorectal cancer.^37^ This is consistent with recent literature.^44^

This review found evidence of UWL as a symptom of various serious conditions. While cancer should be considered, especially in those over 60, other common disorders, particularly in those under 60, should not be overlooked. Withrow *et al*’s pathway for general investigations in primary care offers a valuable framework, ensuring a comprehensive approach tailored to specific populations (Supplementary Table S6).^8^ Another source of evidence to inform the selection of investigations for patients with unspecific symptoms, particularly UWL, is Rapid Diagnostic Centres in the UK. Rapid Diagnostic Centres, designed to expedite cancer detection in patients with non-specific symptoms, highlight weight loss as a common reason for referral. Preliminary evaluations show a significant percentage diagnosed with non-cancer conditions, emphasising the need for comprehensive investigations.^45^ In a preliminary evaluation, 66% of patients were referred due to weight loss (the most common reason for referral in this cohort). Of all patients referred, 8% had a cancer diagnosis and over 50% were diagnosed with non-neoplastic conditions.^46^ In another study, cancer was identified in 7% of patients and 35% of patients were diagnosed with serious non-neoplastic conditions.^47^

### Implications for research and practice

Given the nature of primary care and the challenges of recognising non-specific symptoms like UWL, CDSS could play a pivotal role in early recognition and assessment.^48^ CDSS utilise algorithms aligning scientific literature and clinical guidelines with patient information in EMRs to provide specific recommendations for clinicians.^49^ A systematic review suggests CDSS’s potential to improve cancer referrals and reduce the time to diagnosis. However, barriers to implementation, such as workflow integration, need to be addressed.^48^ Our review serves as a foundation for future research focusing on translating knowledge into actionable recommendations, potentially through tailored CDSS and guideline applications targeting unspecific symptoms like UWL.

^39^ ^15^

This scoping review provided strong evidence of UWL’s association with cancer risk in primary care. Evidence suggests that there is greater than 3% risk of cancer and other serious diseases in patients presenting with UWL, 3% being the recommended threshold for investigation, and we highlighted studies using data to provide tailored recommendation for patients according to age, sex, and the probability of diagnosis. New guidelines should be updated to include this information for investigation.

The pathway to implementation can be difficult, and digital technologies, such as a CDSS can help the translation pathway into practice. We will use these results to inform a CDSS to identify UWL patients at risk of cancer in Australian general practices in 2024.^50^

## Data

All data generated or analysed during this study are included in this published article and its supplementary information files.
